# Amplitude of Lower Limb Muscle Activation in Different Phases of the Illinois Test in Parkinson’s Disease Patients: A Pilot Study

**DOI:** 10.3390/jcm13195792

**Published:** 2024-09-28

**Authors:** Carlos Villarón-Casales, Nieves de Bernardo, Jorge Alarcón-Jiménez, Daniel López-Malo, Belén Proaño, Julio Martín-Ruiz, José Enrique de la Rubia Ortí

**Affiliations:** 1 Biomechanics and Physiotherapy in Sports (BIOCAPS), Faculty of Health Sciences, European University of Valencia, 46001 Valencia, Spain; carlosalberto.villaron@universidadeuropea.es (C.V.-C.); daniel.lopez2@universidadeuropea.es (D.L.-M.); 2 Department of Physiotherapy, Catholic University of Valencia, 46900 Valencia, Spain; nieves.debernardo@ucv.es; 3 Department of Nursing, Catholic University of Valencia, 46001 Valencia, Spain; be.proano@ucv.es (B.P.); joseenrique.delarubi@ucv.es (J.E.d.l.R.O.); 4 Department of Health and Functional Assessment, Catholic University of Valencia, 46900 Valencia, Spain

**Keywords:** Parkinson’s disease, gait, Illinois agility test, electromyography, lower limb

## Abstract

**Background:** Parkinson’s disease (PD) is a neurodegenerative disorder with high prevalence in men and is characterized by symptoms such as tremors and gait difficulties. This study aimed to determine muscle activation in patients with PD by considering sex differences. **Methods:** This pilot study used analytical, quantitative, observational, and case-control methods. Surface electromyography was used to assess muscle activity during a variant of the Illinois agility test. The study population comprised an experimental group of patients with PD (N = 30) and a control group of healthy individuals without the disease (N = 10). **Results:** The Illinois agility test revealed significant differences in completion times between the groups. The Parkinson’s disease group took longer overall (*p* = 0.004), especially for standing up (*p* < 0.001) and sitting down (*p* = 0.002), than the control group. In the control group, sex influenced gastrocnemius muscle activation, with women showing higher activation (r_s_ = −0.87). Women also had greater rectus femoris activation during standing and sitting, with higher activation on the right side when standing (r_s_ = −0.66) and the left side when sitting (r_s_ = −0.87). In the control group, men exhibited greater activation of the right biceps femoris (r_s_ = 0.87). However, in the Parkinson’s disease group, sex did not affect muscle activation. **Conclusions:** Patients with Parkinson’s showed lower muscle activation than healthy individuals while standing up, sitting down, and walking.

## 1. Introduction

Parkinson’s disease (PD) is a progressive neurodegenerative disorder that affects approximately 10 million individuals globally, with a high prevalence in men [1]. The disease is characterized by movement disorders resulting from motor abnormalities, including tremors, bradykinesia, rigidity, muscle cramps and the inclusion of sarcopenia within these symptoms is currently being debated [2,3]. Among the techniques utilized to detect these symptoms (electroencephalography, electromyography, long latency reflexes, cutaneous silent period, studies of cortical excitability [4], surface electromyography (EMG) has been extensively employed in PD to identify the appropriate synergies, and to detect possible locomotor deficits in patients [5,6]. For instance, measuring plantar flexor activation during stance, which is most relevant during this phase (and not during the swing phase), has proven to be a valuable approach [7].

Among the strategies employed to address this disease are functional approaches, such as corrections in subcortical circuits [8] in order to favor postural tasks, which have demonstrated that deep brain stimulation contributes to enhanced agility [9] and motor coordination, reflected in general dynamic actions [10,11], can be identified and used for the early detection of postural instability [7], although there is no clear consensus regarding the generation of coactivations between muscle groups [7]. In addition, locomotion-related strategies aim to identify neuromuscular patterns during gait [12,13]. Finally, tasks focusing on manipulative actions or tremors have demonstrated that appropriate cutaneous stimulation reduces reaction times by enhancing muscular synergies [14,15].

Rehabilitation in this patients has been shown to benefit from the induction of transcranial stimulation, which is associated with the normalization of cortical excitability. This is a fundamental aspect of the homeostatic plasticity [16]. When transcranial stimulation is used at high frequencies, improvements in speed, symmetry, and motor function of the lower limbs have been reported; however, there have been no differences in corticomotor excitability or the functional asymmetry ratio of the brain, as evidenced by both experimental and control groups [17].

A comparative analysis of the effects of tasks requiring voluntary strength on muscle activation in healthy subjects and those with Parkinson’s disease revealed significantly lower muscle activation during the various types of contractions in subjects with Parkinson’s disease compared to healthy individuals [18]. This observation indicates a reduction in strength and muscle efficiency, which has a negative impact on walking speed and is a reliable indicator of PD evolution [19]. The diminished physical function observed in subjects with mild Parkinson’s disease may be attributed to the disease itself and muscle atrophy resulting from physical inactivity rather than reduced muscle activation [20]. In mobility tests, a slower velocity was exhibited in the up-and-go test, and a shorter distance was covered in the 6 min walk test, also monitored with IMU systems [21].

Specifically, alterations in locomotion can be attributed to muscular patterns that encompass a multitude of synergies. Among the assessment tests, variants of a test, such as the Illinois agility test (IAT), provide information about locomotion in situations such as changes in direction or orientation, as well as evaluating parameters such as speed, acceleration, and deceleration [22,23]. This test is frequently used in neurodegenerative diseases [24]. The functional characteristics of the musculoskeletal system can be demonstrated, for example, by examining how the stimulation of peripheral muscles, such as the tibialis anterior or gastrocnemius, affects muscle activation during gait. This can result in enhanced efficiency without any alteration to the kinematics [25].

The improvement in dynamic balance, which is a key aspect of gait, has been shown to result in notable improvements in patients with mild to moderate symptoms [26]. A few other interventions should be noted, including rowing movements, agility circuits, adapted boxing tasks, and lunge exercises [27]. In general, symmetrical tasks are appropriate for better motor control and contribute to an improvement in the quality of life of a patient [28,29].

Considering the existing literature on this topic, the main objective of this study was to measure the muscle activation of Parkinson’s disease patients (experimental group) and compare it with a group of healthy individuals (control group) during the IAT. This analysis considered the distinct components of the test, including standing up, walking back and forth, sitting down, and the sex of the participants. The main hypothesis of the study is that the PD group will register higher time values in all phases of the test than the control group, as well as less compensated muscle activation.

The findings produced will be of great relevance to more precisely identify the activation sequence in each phase of the test, being able to detect compensatory patterns that will allow the individualization of appropriate therapies for each case.

## 2. Materials and Methods

### 2.1. Study Design and Participants

This pilot study was a cross-sectional, observational, descriptive study, with a group of individuals diagnosed with PD and a control group comprising healthy participants.

The Valencian Community “Parkinson’s Association Valencia” (Spain) was asked to inform its members of the nature of this research. Volunteers contacted the principal investigator of the study, who provided them with a patient information sheet and informed consent form, which were duly signed by all patients. After obtaining informed consent, a series of selection criteria were applied. The inclusion criteria were as follows: (i) patients over the age of 18 years diagnosed with Parkinson’s and (ii) patients with mild disease (Hoehn and Yahr stage 2 or lower). The exclusion criteria were as follows: (i) severe psychotic symptoms (score > 2 on item 1.2 of the Unified Parkinson’s Disease Rating Scale of the International Parkinson and Movement Disorder Society) or (ii) a clinical diagnosis of dementia.

The control group of individuals without the disease was selected according to the following inclusion criteria: (i) patients of both sexes over the age of 18 years, and (ii) acceptance of participation in the study by signing an informed consent form. Exclusion criteria included: (i) participation in any other trial or in the 4 weeks prior to inclusion, (ii) people with evidence of dementia or diagnosed with another disease that causes alterations in motor activity, and (iii) people with dependency or alcohol or drug abuse.

### 2.2. Electrical Activity Registration

The subject was placed in a seated position, and the area intended for electrode placement was dried from sweat and cleaned with 96% ethyl alcohol. The area was shaved to remove any potential interference due to impedance. The electrodes were placed bilaterally in channels 1 and 2 of the anterior rectus femoris (ARF), which is the central ventral portion of the femur, in a longitudinal position. Channels 3 and 4 were placed obliquely in the biceps femoris (BCF): the dorsal part of the distal third of the femur. Channels 5 and 6 in the anterior tibialis (ATB) were located 4 cm below the head of the fibula in a longitudinal position relative to it. Finally, channels 7 and 8 in the medial gastrocnemius (GNM) were placed in the most prominent part of the muscle belly in a longitudinal position, with the knee extended.

In all cases, the two electrodes were positioned with a maximum separation of 2 cm in the muscle belly. The electrode model used was a 30 mm Lessa Pediatric Electrode (Lessa, AB Medica Group, Barcelona, Spain). Electrode placement was in accordance with the guidelines set forth by the Surface EMG for non-invasive assessment of muscles project (SENIAM) [30] and Criswell [31].

A BTS FREEEMG model device (BTS Bioengineering, Milano, Italy) was used for electromyographic recording. The sampling frequency was 1 KHz, and each stored record had a duration of 60 s. Following data acquisition, the data were saved on a hard drive for protection and subsequently analyzed in files with the extension. The emt and units are in millivolts.

A specific program, MATLAB (R2021a) (Mathworks Inc., Natick, MA, USA), was employed for signal analysis. First, a fourth-order Butterworth bandpass filter was applied between 20 and 400 Hz to filter the signal and the Root Mean Square (RMS) was obtained by dividing the measurement section by 100 points. The units of measurement were converted from millivolts (mV) to microvolts (μV). Subsequently, the total test section was segmented by collecting the entire test dataset, excluding the initial and final sections that did not exhibit any activity.

Smoothed data of the average and maximum amplitudes of the complete signal were extracted using the smoothing function, enabling a comparative analysis of the two records. The test was divided into three sections for analysis: (1) the period from when the subject was sitting until standing up, (2) the activation peaks of the first three steps on the way, and the last three steps on the way back, with the objective of assessing ambulation with the development of the findpeak function. The intermediate section was excluded when a turn occurred that altered the gait. Finally, (3) the action of returning to a seated position was considered. In addition to the average signal in μV across different sections, the time in ms in each section was calculated (getting up, standing, sitting, and total time).

### 2.3. Statistical Analysis

IBM SPSS Statistics software version 23 (SPSS Inc., Chicago, IL, USA) was used for all the analyses. Qualitative variables are described as proportions (%), and quantitative variables are described as mean (M) with standard deviation (SD) and median (Md) with interquartile range (IQR). The normality of the data was evaluated using the Shapiro–Wilk test. To assess the differences between the groups, namely healthy controls and PD patients, the Student’s *t*-test for independent variables was used when the data were normally distributed, whereas the Mann–Whitney U test was used when the data were not normally distributed. Spearman’s correlation coefficient was used to determine the influence of age and sex on both groups. The significance level for all analyses was set at α = 0.05.

### 2.4. Ethical Considerations

This study was conducted in accordance with the Declaration of Helsinki and approved by the Clinical Research Ethics Committee, CEIM 2018/0022 (23/08/2018) of the Hospital Universitario y Politécnico de la Fe de Valencia. The authors adhered to Good Clinical Practice (GCP) standards (CPMP/ICH/135/95) [32] and current legislation. The patients enrolled in the study signed an informed consent form after receiving comprehensive information about the procedures and nature of the study.

## 3. Results

The study sample consisted of 10 healthy participants in the control group and 30 patients with PD disease. The mean age of the control group was 48 ± 9 years, with 50% of the patients being men and women. The mean age of the patients with PD was 55 ± 15 years and 77% were male.

**Figure 1:**
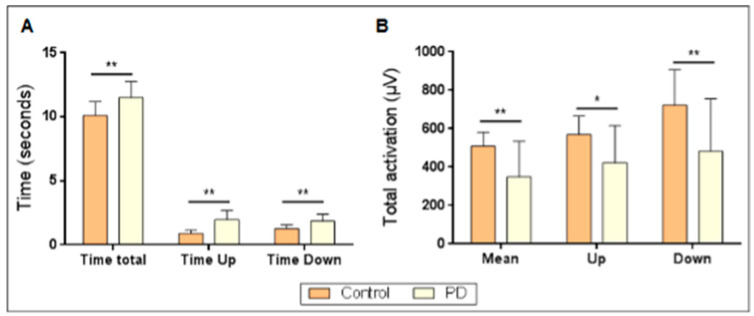
Global performance in the Illinois agility test (IAT) for control and Parkinson’s disease (PD) groups. (**A**) Time required for completing the test: total time spent standing up and sitting down. (**B**) Total activation achieved with the eight muscles during the complete test (mean) when standing up and sitting down. * *p* < 0.05; ** *p* < 0.001.

IAT revealed significant differences in the time required for each group to complete the test (Figure 1A); The group of patients with PD exhibited a longer overall completion time for the entire test (*p* = 0.004), as well as for standing up (*p* < 0.001) and sitting down (*p* = 0.002). The greatest difference was observed in the time required to get up, with the PD group exhibiting a two-fold slower rate than the control group (Figure 1A). Moreover, the total activation achieved by the eight muscles in the test (Figure 1B) also demonstrated statistically significant differences between the groups, with higher mean values observed in the control group for both the global average activation throughout the entire test and the maximum activation achieved, as well as the specific mean activation when participants assumed either a seated or a standing up position. In all the cases, the observed difference exceeded 100 µV.

Table 1 presents the total activation values for each group while standing and sitting. Regarding mean activation during the test development phase, significant differences were observed between the groups in the four muscles, particularly on the right side. For ARF, BCF, and GNM, the control group exhibited greater activation than the PD group exclusively on this side. The control group exhibited greater activation of the ATB muscle on both sides. The differences in ATB-R (*p* = 0.0003) and right medial gastrocnemius (GNM-R) (*p* = 0.0007) were especially striking, with differences of 61 µV and 54 µV, respectively, representing more than twice the activation in the control group.

Upon analysis of muscle activation, when the participants stood up during the test, significant differences were observed on both sides for the ARF and ATB, with lower activation in patients with PD. The greatest difference was found in the right anterior tibialis (ATB-R), where the activation for the patients reached 51 µV, that is, a 3.5-fold reduction compared to the control group. Furthermore, the activation of the right biceps femoris (BCF-R) was significantly diminished in patients with PD. In contrast, the left gastrocnemius (GNM-L), exhibited a fourfold increase in activation upon standing up in the PD group relative to the control group, with mean values of 126.6 µV and 31.6 µV, respectively.

The behavior of the muscles when the participants sat (Table 1) was similar to that found when the patients stood, with greater means in the ARF and ATB muscles in the control group for both sides, although the greatest difference was found for the right side, with triple and double activation in the control group for the ATB-R and the right anterior rectus femoris (ARF-R), respectively. The BCF also showed greater activation in the control group, but only on the right side. Similarly, the GNM-L was highly activated in the PD group (*p* = 0.0004), with almost triple activation compared with that in the control group.

The effect sizes for each variable are also reported in Table 1, with middle to strong effect sizes (r > 0.5) for the differences in ATB-R and GNM-R in the mean activation; ARF-R and ATB-R during the standing-up trial; and ARF-R and ATB-R during the sitting-down trial. The majority of statistically significant differences had a moderate effect size (0.3 < r < 0.45).

Finally, regarding the impact of demographic variables such as age and sex on IAT performance, the control group showed the influence of sex on the mean activation and maximum activation of the GNM on both sides (r_s_ = −0.87), indicating that women demonstrated greater activation in these muscles. Furthermore, women also exhibited greater activation of the ARF on the right side when standing up (r_s_ = −0.66) and the left side was more active when sitting down (r_s_ = −0.87). Male participants exhibited greater activation levels, specifically maximum activation of the BCF on the right side (r = 0.87). Conversely, age demonstrated a significant impact on only two muscles on the right side: the ARF (r_s_ = −0.72) and TBA (r_s_ = −0.70). In these instances, the association was negative and robust, indicating that the older participants achieved lower maximum activation levels.

In contrast, no muscle activation was associated with sex in the PD group. Regarding age, a significant positive association was observed only with activation of the right TBA, as indicated by the mean global activation and maximum activation when standing up and sitting. This suggests that older patients exhibit greater activation of this muscle. All correlation coefficients can be found in Appendix A. Supplementary information can be found in Table S1 (supplementary).

## 4. Discussion

Periodic assessment tests are effective for monitoring disease processes. Tests such as the 6 min walk test and the up-and-go test have been demonstrated to be highly relevant in the case of Parkinson’s disease, as they are characterized by being functional and transferable to real-life situations, as has already been referenced in the literature [22,23].

One of the earliest differentiating factors between healthy participants and those with disease is the duration of ambulation tasks. In this study, we expanded upon previous research, such as that of Kim et al. [20], in which only total test time was measured. In this study, all segments of the test were additionally calculated, including the time taken to get up (*p* = 0.01), sit down (*p* = 0.002), and the total time (*p* = 0.0041). The observed differences were significant, with Parkinson’s patients consistently exhibiting longer times, indicating a neurodegenerative impact on the general dynamic coordination of low complexity.

In the case of the bilateral muscle-activation data collected, it is noteworthy that, during ambulation, in the control group (healthy people), the muscles with the highest average activation, and with the greatest percentage contribution to the test are the ATB (106.72 ± 26.41 right and 87.02 ± 20.62 left), and the GNM (82.86 ± 36.63 right and 73.87 ± 21.98 left), which typically play a leading role in propulsive actions [33], such as those observed in this test. These actions consistently exhibited similar means for both sides, with greater activation on the right side. These data on normality are in contrast with those of the experimental group, in which this impulse-generating activity was absent, particularly in the gastrocnemius and tibialis anterior muscles. This contributes to the phenomenon of lame gait, which is distinct from that observed in the healthy subjects (*p* = 0.0004). The only effect observed in the control group was sex-related, which is consistent with the normality reported in previous studies [34]. This suggests that there may be alterations in patients with PD as women in the control group exhibited greater global activation of the GNM on both sides, a pattern not observed in the PD group.

Activity in the thigh muscles was lower in the PD group than in the control group during ambulation (*p* = 0.014), standing up (*p* = 0.013), and sitting down (*p* = 0.008). Furthermore, these findings frequently manifest bilaterally, indicating a deficit in driving and the necessity for augmented activation of the GNM. This compensatory phenomenon has been previously documented during muscular actions in the context of neurodegenerative diseases [35].

This synergistic muscular pattern, for which the literature should provide more evidence [36], was repeated at the beginning and end of the test, highlighting the left gastrocnemius. When standing up, the activation was four times greater than that in the control group and three times greater when returning to a sitting position (*p* = 0.004 in both cases); for these reasons, the hypothesis has been confirmed both in terms of time and muscle activation values. These findings are relevant to the formulation of fall risk prevention strategies in the context of both standing and sitting, as well as to delineate the necessity for the implementation of a targeted therapeutic approach, as postulated by Tutus and Ozdemir [33]. They caution against the potential necessity of intervention for gastrocnemius spasticity to enhance the postural control of gait when such conditions arise. Despite these findings, it is necessary to remember, as Ghislieri et al. [37] indicate, the loss of synergy that is common in Parkinson’s disease is accentuated in stabilization and dynamic postural control tasks. Therefore, within any therapeutic strategy, the conditioning of the native musculature responsible for these actions is unquestionable, even in the aquatic environment, where this activation pattern can also be improved [38]. In fact, devices such as Equistasi^®^ focus on improving global activation for better postural control, showing that this trend seems to be the line of work of the future [39,40].

### Limitations of the Study

Despite the findings presented in this study, there are several limitations inherent in a pilot study. The most important ones are the small sample size and the heterogeneity of the control and PD groups in the sociodemographic variables. However, the statistical power even with this sample size and the 3:1 proportions remains at β = 0.8, for strong effect sizes. On the other hand, the group heterogeneity might be due to the convenience sampling procedure to enroll control subjects, and it should be taken into consideration when extrapolating conclusions. Thus, this pilot study can give insight to possible differences in muscular activation patterns, although further studies would benefit from a larger sample size and better matching with controls, as well as the possibility of implementing blinding procedures during the analysis. It also would be valuable to correlate these muscular measurements with the progression of the disease and functionality of the patients using standardized scales.

## 5. Conclusions

Following the presentation of the primary findings, the study’s conclusion, in response to the stated objective, is that patients with Parkinson’s demonstrate a reduction in cerebral activation when performing activities such as standing, sitting, and walking compared to healthy individuals. It is imperative to highlight the significance of fall prevention and the necessity of developing efficacious therapies to enhance agility in the context of daily functional activities. To this end, it is essential to understand the compensatory mechanisms of gait in patients with Parkinson’s, as this will facilitate the implementation of biomechanical enhancements and the design of an efficacious individualized treatment plan. Therefore, from a neurological point of view, the work of muscle chains and their interrelation could be appropriate. Moreover, it is important to implement a fall risk prevention program that enhances functional capacity in activities, such as travel, which is essential for maintaining autonomy. In summary, the speed of execution, symmetry observed, and proportion of contribution from each muscle group can facilitate a more comprehensive understanding of the gait pattern and access to targeted therapies that achieve functional improvement in patients with Parkinson’s disease.

## 6. Future Perspectives

In the specific field of movement analysis, one of the keys can be found in the importance of interdisciplinary work, in which medicine, physiotherapy, sport sciences, and those derived from biomechanics, to design comprehensive studies to collect objective data, such as optimizing specific therapies.

This aspect can be optimized by implementing techniques that include AI and machine learning (ML) to identify and classify neuroimaging methods, gait abnormalities or speech recordings [41], which have demonstrated greater diagnostic accuracy and improved decision-making on the therapies to be applied [42].

## Figures and Tables

**Table 1:** Mean activation for each group in the overall IAT and each section.

	Control (N = 10)				PD (N = 30)					
	M	SD	Md	IQR	M	SD	Md	IQR	*p*-Value	r
ARF-R (MA)	38.11	14.96	33.2	8.3	23.62	19.78	15.6	24.9	0.014	−0.39
ARF-L (MA)	32.87	9.32	30.4	14.4	28.50	20.91	22.3	29.4	0.150	−0.23
BCF-R (MA)	46.52	12.70	45.9	17.5	30.42	23.81	25.4	27.5	0.005	−0.44
BCF-L (MA)	41.02	11.80	37.4	18.6	49.66	38.44	48.5	40.8	0.574	−0.09
ATB-R (MA)	106.72	26.41	103.5	34.7	44.84	57.22	15.8	63.4	<0.001	−0.58
ATB-L (MA)	87.02	20.62	85.2	11.9	65.46	49.61	46.5	66.6	0.042	−0.32
GNM-R (MA)	82.86	36.63	71.4	74.5	28.23	30.70	10.0	50.4	<0.001	−0.54
GNM-L (MA)	73.87	21.98	71.8	39.5	82.42	72.36	57.7	85.4	0.472	−0.11
ARF-R (Up)	69.45	25.43	67.7	46.8	26.67	21.52	20.5	28.2	<0.001	−0.61
ARF-L (Up)	62.57	26.48	53.1	34.3	40.74	41.96	24.7	46.4	0.013	−0.39
BCF-R (Up)	37.77	15.89	37.2	26.7	27.46	29.66	19.2	17.4	0.013	−0.39
BCF-L (Up)	27.92	11.82	23.0	14.7	46.38	38.88	35.8	39.9	0.118	−0.25
ATB-R (Up)	180.24	73.47	161.8	57.7	50.99	61.88	19.7	78.2	<0.001	−0.64
ATB-L (Up)	129.89	48.50	123.6	77.0	87.40	68.13	68.2	68.7	0.020	−0.37
GNM-R (Up)	30.39	34.70	20.0	10.2	28.33	33.79	11.8	54.4	0.169	−0.22
GNM-L (Up)	31.59	14.96	32.2	26.2	126.66	129.75	76.7	150.6	0.004	−0.44
ARF-R (Down)	86.83	42.92	78.8	54.9	41.88	57.27	21.4	38.3	<0.001	−0.53
ARF-L (Down)	69.91	24.32	75.2	33.2	38.26	38.25	23.6	35.6	0.008	−0.41
BCF-R (Down)	55.37	39.63	40.5	52.6	32.59	37.12	25.0	19.3	0.008	−0.41
BCF-L (Down)	51.66	61.56	31.0	28.2	53.36	43.29	46.8	38.5	0.365	−0.14
ATB-R (Down)	209.83	62.57	204.3	108.5	67.83	92.70	21.5	88.3	<0.001	−0.59
ATB-L (Down)	165.19	69.11	151.8	149.1	103.70	100.21	61.0	108.5	0.016	−0.38
GNM-R (Down)	45.65	31.65	40.6	32.6	29.09	32.80	11.1	45.6	0.075	−0.28
GNM-L (Down)	38.72	24.04	36.5	32.0	110.20	98.50	181.1	183.5	0.004	−0.31

PD: Parkinson’s disease, IAT: Illinois agility test M: mean, SD: standard deviation, Md: median, IQR: interquartile range, R: right, L: left, ARF: anterior rectus femoris, BCF: biceps femoris, ATB: anterior tibialis, GNM: gastrocnemius; activation was measured in µV. r coefficients calculated for effect size.

## Data Availability

The datasets used and analyzed during the current study are available from the corresponding author upon reasonable request owing to privacy and ethical restrictions.

## Supplementary Materials

The following supporting information can be downloaded at: https://www.mdpi.com/article/10.3390/jcm13195792/s1, Table S1: Effect size and confidence intervals for the mean of the differences of each variable.

## Abbreviations

PD (Parkinson’s disease); EMG (electromyography); IAT (Illinois agility test); ARF (anterior rectus femoris); BCF (biceps femoris); ATB (anterior tibialis); GNM (gastrocnemius); GNM-R (gastrocnemius right or left L); IQR (interquartile range); μV (microvolts); M (mean); SD (standard deviation); Md (median); GCP (good clinical practice); MA (main activation).

## Appendix A

**Table A1:** Additional descriptive data.

	Control					PD			
	Sex		Age			Sex		Age	
	r_s_	*p*-Value	r_s_	*p*-Value		r_s_	*p*-Value	r_s_	*p*-Value
ARF-R (MA)	–0.174	0.631	–0.711 *	0.021	ARF-R (MA)	0.087	0.649	0.257	0.170
ARF-L (MA)	0.244	0.497	–0.571	0.084	ARF-L (MA)	–0.032	0.867	0.361 *	0.050
BCF-R (MA)	0.244	0.497	–0.195	0.590	BCF-R (MA)	0.214	0.256	0.110	0.562
BCF-L (MA)	–0.383	0.275	–0.043	0.907	BCF-L (MA)	0.077	0.684	0.220	0.243
ATB-R (MA)	–0.035	0.924	–0.286	0.424	ATB-R (MA)	0.278	0.137	0.305	0.102
ATB-L (MA)	–0.244	0.497	0.274	0.444	ATB-L (MA)	0.296	0.112	0.404 *	0.027
GNM-R (MA)	–0.870 **	0.001	0.158	0.663	GNM-R (MA)	0.032	0.867	0.155	0.413
GNM-L (MA)	–0.870 **	0.001	0.146	0.688	GNM-L (MA)	–0.032	0.867	–0.015	0.938
ARF-R (Up)	–0.661 *	0.037	–0.267	0.455	ARF-R (Up)	0.114	0.549	0.287	0.123
ARF-L (Up)	–0.453	0.189	–0.419	0.228	ARF-L (Up)	0.041	0.830	0.310	0.095
BCF-R (Up)	–0.174	0.631	0.170	0.638	BCF-R (Up)	0.114	0.549	0.043	0.822
BCF-L (Up)	–0.244	0.497	0.170	0.638	BCF-L (Up)	–0.005	0.981	0.131	0.489
ATB-R (Up)	0.244	0.497	–0.322	0.364	ATB-R (Up)	0.260	0.166	0.261	0.163
ATB-L (Up)	0.592	0.071	0.097	0.789	ATB-L (Up)	0.260	0.166	0.440 *	0.015
GNM-R (Up)	–0.592	0.071	0.176	0.626	GNM-R (Up)	–0.100	0.598	0.075	0.693
GNM-L (Up)	–0.522	0.122	0.316	0.374	GNM-L (Up)	–0.178	0.348	–0.219	0.244
ARF-R (Down)	–0.453	0.189	–0.267	0.455	ARF-R (Down)	0.041	0.830	0.220	0.242
ARF-L (Down)	–0.870 **	0.001	–0.067	0.854	ARF-L (Down)	–0.132	0.487	0.336	0.069
BCF-R (Down)	0.104	0.774	–0.383	0.275	BCF-R (Down)	0.087	0.649	0.096	0.613
BCF-L (Down)	–0.522	0.122	–0.085	0.815	BCF-L (Down)	0.096	0.615	0.151	0.426
ATB-R (Down)	–0.313	0.378	–0.109	0.763	ATB-R (Down)	0.232	0.217	0.298	0.110
ATB-L (Down)	–0.383	0.275	0.328	0.354	ATB-L (Down)	0.159	0.400	0.405 *	0.026
GNM-R (Down)	–0.453	0.189	–0.061	0.868	GNM-R (Down)	–0.005	0.981	0.101	0.596
GNM-L (Down)	–0.035	0.924	0.304	0.393	GNM-L (Down)	–0.141	0.457	–0.067	0.725
ARF-R (Max)	0.035	0.924	–0.723 *	0.018	ARF-R (Max)	0.141	0.457	0.134	0.480
ARF-L (Max)	–0.244	0.497	–0.565	0.089	ARF-L (Max)	0.132	0.487	0.310	0.095
BCF-R (Max)	0.870 **	0.001	–0.292	0.413	BCF-R (Max)	0.178	0.348	0.203	0.282
BCF-L (Max)	–0.174	0.631	0.140	0.700	BCF-L (Max)	0.187	0.323	0.292	0.118
ATB-R (Max)	0.035	0.924	–0.705 *	0.023	ATB-R (Max)	0.250	0.182	0.288	0.123
ATB-L (Max)	0.592	0.071	–0.164	0.650	ATB-L (Max)	0.260	0.166	0.407 *	0.026
GNM-R (Max)	–0.870 **	0.001	0.292	0.413	GNM-R (Max)	0.096	0.615	0.175	0.355
GNM-L (Max)	–0.731 *	0.016	0.006	0.987	GNM-L (Max)	–0.032	0.867	0.041	0.831

PD: Parkinson’s disease, R: right, L: left, ARF: anterior rectus femoris, BCF: biceps femoris, ATB: anterior tibialis, GNM: gastrocnemius, MA: main activation. *: *p* < 0.05. ** *p* < 0.01.
