# Supplementary material for: Amplitude of Lower Limb Muscle Activation in Different Phases of the Illinois Test in Parkinson’s Disease Patients: A Pilot Study
Source: J Clin Med. 2024 Sep 28;13(19):5792. doi: 10.3390/jcm13195792 (PMC11476849; doi:10.3390/jcm13195792)
Supplement: Supplementary file 1 [file jcm-13-05792-s001.zip › jcm-3169010-supplementary.pdf]

Supplementary material

Table S1. Effect size and confidence intervals for the mean of the differences of each variable.

	Test	Z	r	d	p-value	MD	IC
ARF-R (MA)	u	-2.436	-0.39	-0.83	0.014	14.50	0.64, 28.36
ARF-L (MA)	u	-1.437	-0.23	-0.47	0.150	4.37	-9.55, 18.28
BCF-R (MA)	u	-2.780	-0.44	-0.98	0.005	16.09	0.05, 32.13
BCF-L (MA)	u	-.562	-0.09	-0.18	0.574	-8.64	-33.82, 16.54
ATB-R (MA)	u	-3.654	-0.58	-1.42	<0.001	61.88	23.72, 100.03
ATB-L (MA)	u	-2.030	-0.32	-0.68	0.042	21.57	-11.32, 54.45
GNM-R (MA)	u	-3.405	-0.54	-1.28	<0.001	54.63	30.82, 78.43
GNM-L (MA)	u	-.718	-0.11	-0.23	0.472	-8.55	-55.94, 38.84
ARF-R (Up)	u	-3.842	-0.61	-1.53	<0.001	42.78	26.15, 59.42
ARF-L (Up)	u	-2.468	-0.39	-0.85	0.013	21.82	-6.9, 50.54
BCF-R (Up)	u	-2.468	-0.39	-0.85	0.013	10.31	-9.68, 30.3
BCF-L (Up)	u	-1.562	-0.25	-0.51	0.118	-18.46	-43.93, 7
ATB-R (Up)	u	-4.029	-0.64	-1.65	<0.001	129.24	81.34, 177.15
ATB-L (Up)	u	-2.311	-0.37	-0.79	0.020	42.49	-4.83, 89.82
GNM-R (Up)	u	-1.374	-0.22	-0.45	0.169	2.06	-23.08, 27.2
GNM-L (Up)	u	-2.811	-0.44	-0.99	0.004	-95.07	-179.03, -11.12
ARF-R (Down)	u	-3.373	-0.53	-1.26	<0.001	44.95	4.87, 85.02
ARF-L (Down)	u	-2.624	-0.41	-0.91	0.008	31.64	5.44, 57.84
BCF-R (Down)	u	-2.624	-0.41	-0.91	0.008	22.78	-5.11, 50.67
BCF-L (Down)	u	-.906	-0.14	-0.29	0.365	-1.70	-37.37, 33.96
ATB-R (Down)	u	-3.717	-0.59	-1.45	<0.001	142.00	78.05, 205.96
ATB-L (Down)	u	-2.405	-0.38	-0.82	0.016	61.49	-7.83, 130.81
GNM-R (Down)	u	-1.780	-0.28	-0.59	0.075	16.56	-7.49, 40.61
GNM-L (Down)	u	-1.937	-0.31	-0.64	0.004	-85.80	-166.71, -4.9
Total activation: Mean	t	2.599	0.39	0.84	0.0005	155.84	73.34, 238.34
Total activation: Max	u	-3.436	-0.54	-1.29	0.0006	791.66	507.7, 1075.62
Total activation: Up	t	2.037	0.31	0.66	0.0486	135.18	38.54, 231.83
Total activation: Down	u	-2.86	-0.45	-1.01	0.0042	240.04	80.42, 399.66

Test: Mann Whitney's U (non-normal distribution) or student's t test (normal distribution). For effect size: Pearson's r coefficient (r) and Cohen's d (d). MD: mean of the differences. CI: 95% confidence interval.
